# TEMPOL has limited protective effects on renal oxygenation and hemodynamics but reduces kidney damage and inflammation in a rat model of renal ischemia/reperfusion by aortic clamping

**Published:** 2015-09-30

**Authors:** Bulent Ergin, Rick Bezemer, Asli Kandil, Cihan Demirci-Tansel, Can Ince

**Affiliations:** 1 Department of Translational Physiology, Academic Medical Center, University of Amsterdam, Amsterdam, The Netherlands; 2 Department of Biology, Faculty of Science, Istanbul University, Vezneciler, Istanbul, Turkey

**Keywords:** oxidative stress, antioxidants, acute kidney injury, cortical and medullar microcirculation, sterile immune response, systemic and renal hemodynamics, microvascular oxygenation

## Abstract

**Background:**

Renal ischemia-reperfusion (I/R) is a common clinical complication in critically ill patients that is associated with considerable morbidity and mortality. Renal I/R is a major cause of acute kidney injury (AKI) resulting from I/R-induced oxidative stress, sterile inflammation, and microcirculatory perfusion defects, which can be ameliorated with the superoxide scavenger TEMPOL. The most common cause of AKI in the clinical setting is aortic surgery with suprarenal aortic clamping. The protective effect of TEMPOL in aortic clamping-induced renal I/R has not been studied before.

**Aim:**

To evaluate the protective effects of TEMPOL on oxidative stress, inflammation, tissue injury, and renal hemodynamics and oxygenation in a clinically representative rat model of I/R using aortic cross-clamping.

**Methods:**

Animals (N = 24) were either sham-operated or subjected to ischemia (30 min) and 90-min reperfusion, with or without TEMPOL treatment (15 min before ischemia and during entire reperfusion phase, 200 μmol/kg/h). Systemic and renal hemodynamics, renal oxygenation, and blood gas values were determined at 15 min and 90 min of reperfusion. At 90-min reperfusion, iNOS, inflammation (IL-6, MPO), oxidative stress (MDA), and tissue damage (NGAL, L-FABP) were determined in tissue biopsies.

**Results:**

TEMPOL administration at a cumulative dose of 400 μmol/kg conferred a protective effect on AKI in terms of reducing renal damage, inflammation, and iNOS activation. With respect to renal hemodynamics and oxygenation, TEMPOL only reduced renal vascular resistance to near-baseline levels at both reperfusion time points and partially ameliorated the I/R-induced drop microvascular partial tension of oxygen at 90 min reperfusion. Also, TEMPOL alleviated the I/R-induced metabolic acidosis. However, TEMPOL exerted no restorative effect in terms of the severely reduced mean arterial pressure, renal blood flow, and renal oxygen delivery and consumption. The renal oxygen extraction ratio remained unchanged during the 90-min reperfusion phase. Kidneys in all groups were anuric throughout the experiment.

**Conclusions:**

This clinically representative renal I/R model, which entails both renal I/R and hind limb I/R as opposed to the standardly used renal I/R model that employs renal artery clamping, resulted in relatively moderate *direct* AKI. The damage was exacerbated by the perturbed systemic hemodynamics and metabolic acidosis as a result of the hind limb I/R. TEMPOL partially intervened in the factors that led to AKI as well as renal microvascular partial tension of oxygen and metabolic acidosis. However, more effective interventions should be devised for the mean arterial pressure drop (i.e., anuria) associated with aortic clamping and for restoring other critical renal hemodynamic and oxygenation parameters in order to improve post-I/R renal function.

**Relevance for patients:**

TEMPOL is a promising compound that has been shown to protect kidneys from I/R damage, which is relevant in kidney transplantation, pancreas transplantation, and aortic aneurysm repair in kidney transplant patients. This study suggests that intervening with TEMPOL is not sufficient to ensure optimal clinical outcome in patients that have undergone aortic clamping and that more effective interventions should be investigated.

## 1. Introduction

Renal ischemia/reperfusion (I/R) is a common clinical complication in critically ill patients that leads to a high incidence of morbidity and mortality [1]. Renal I/R is a major cause of acute kidney injury (AKI) [2]. Although the etiology of AKI is multifarious [3], the most common cause of AKI in the clinical setting is a suprarenal aortic clamping [4], which is performed in procedures such as kidney transplantation [5], pancreas transplantation [6], and aortic aneurysm repair [7].

**Figure 1.**
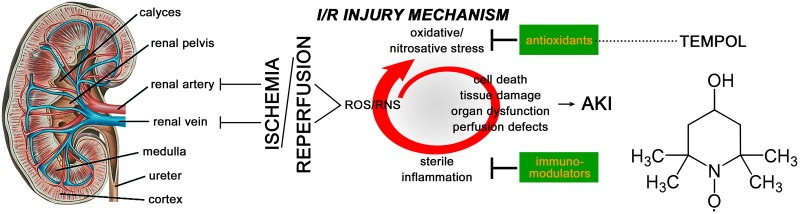
Gross anatomy of the kidney, the general mechanisms of renal ischemia/reperfusion (I/R) injury, and the most plausible pharmacological intervention sites for the amelioration of renal I/R injury. Cessation of blood supply to the kidney causes ischemia that, when ensued by reperfusion, results in the production of reactive oxygen and nitrogen species (ROS/RNS, respectively). The excessive generation of ROS/RNS leads to oxidative/nitrosative stress, the oxidative/nitrosative modification of biomolecules, and ultimately cell death, acute kidney injury (AKI), and renal (microvascular) dysfunction. Dead and dying cells release self-antigens called damage-associated molecular patterns (DAMPs) that chemotactically recruit and activate immune cells (neutrophils and monocytes/macrophages) in the injured kidney. In the absence of pathogens (i.e., sterile inflammation), the activated immune cells generate ROS/RNS that are directed at renal vascular and parenchymal cells, exacerbating the prevailing oxidative/nitrosative stress and corollary AKI. Accordingly, two plausible types of interventions entail (1) the administration of antioxidants (e.g., TEMPOL) or agonists of endogenous antioxidants to reduce the extent of oxidative stress and (2) immunomodulatory agents to downscale the pro-inflammatory response and associated ROS/RNS generation.

The general pathogenic mechanism underlying I/R- mediated AKI is summarized in Figure 1 [2, 8-11]. The development of AKI entails a vicious cycle involving reactive oxygen and nitrogen species (ROS and RNS, respectively), parenchymal cell death and structural damage, and sterile inflammation (Figure 1). In addition, ROS and RNS contribute to microvascular dysfunction and debilitated microvascular oxygenation in I/R-subjected kidneys [2, 11]. Rodent I/R AKI models revealed that I/R-mediated endothelial injury [12], characterized by e.g., glycocalyx degradation [13, 14], microvascular thrombosis [15], and endothelial activation [8], affects the peritubular microcirculation (e.g., perfusion defects [16, 17]) and culminates in organ dysfunction [18]. These effects have also been reported for I/R AKI in patients [19, 20]. Moreover, perturbations in local oxygenation due to microvascular perfusion defects [16, 17] translate to debilitated mitochondrial function and energy metabolism that in turn contribute to diminished kidney viability, function, and restorative capacity [8, 21]. Corroboratively, Funk et al. demonstrated that AKI causes mitochondrial dysfunction in the renal cortex and that this state correlates with sustained tubular damage [22]. Taken altogether, oxygenation and oxidative stress are intricately related during renal I/R and lie at the basis of AKI [8].

The biological trigger of oxidative/nitrosative stress is superoxide, which is generated in mitochondria at low levels under normal physiological conditions [23] but hyperproduced following I/R in both endothelial cell [24] and renal mitochondria [25]. Intravascular sources of superoxide during I/R include xanthine oxidase and NADPH oxidase (NOX) in endothelial cells, leukocytes, and platelets [10], whereby NOX1 [26], NOX2 [27], and NOX4 [28] have been implicated in renal I/R. Although relatively innocuous itself, superoxide gives rise to more toxic secondary and tertiary reactive intermediates [29] that form from the superoxide dismutase-catalyzed end product hydrogen peroxide [30-32]. Superoxide also reacts with NO that is extensively produced during renal I/R [33] to form peroxynitrite [34, 35], which oxidatively modifies proteins, DNA bases, and lipids by nitration, rendering the biomolecules dysfunctional. The reaction between NO and superoxide operates at a diffusion-controlled rate and therefore predominates over virtually all other competing reactions. Furthermore, peroxynitrite perturbs electron transport chain functionality (i.e., aerobic respiration), inhibits membrane Na^+^/K^+^-ATPase activity, and activates pro-apoptotic enzymes [36]. Superoxide therefore constitutes an important therapeutic target in I/R AKI [37].

Pharmacological interventions aimed at reducing the deleterious (downstream) effects of superoxide overproduction by the administration of antioxidants or agonists of endogenous antioxidants [38] (Figure 1) have proven beneficial and effective against I/R injury in the heart [39], liver [40], brain [41, 42], intestines [43], lungs [44], skin [45], and kidney [46], albeit the protective effects are not always ubiquitous [47]. With respect to the kidneys, experimental studies have demonstrated that membrane-permeable, low molecular weight SOD mimetics may improve post-I/R outcomes due to their beneficial effects on ROS scavenging and mitochondrial metabolism [48-50]. TEMPOL (4-hydroxy-2,2,6,6-tetramethyl piperidinoxyl) is a membrane-permeable, metal-independent SOD mimetic with specific reactivity towards superoxide [51] and hence capable of intervening early in the injurious redox chain and renal pathogenesis (Figure 1). Also, TEMPOL has been shown to cause dilation of I/R-constricted (inflamed) coronary blood vessels [52] and retinal arterioles [53]. Accordingly, numerous studies have shown that TEMPOL reduces I/R AKI in different species, including rats [54].

The antioxidant [55], immunosuppressive [56, 57], and AKIameliorating effects of TEMPOL are well-established in I/R AKI. Conversely, relatively little information is available in terms of renal oxygenation, which is critical for renal function [8]. In a study that was conducted in parallel to the work presented here [58], the protective effects of TEMPOL on (micro)vascular hemodynamics and oxygenation were demonstrated in a setting of renal I/R induced by renal artery clamping (30 min ischemia, 90 min reperfusion). However, this procedure is not representative for the clinical situation where AKI is mainly induced by aortic clamping [4], affecting more than just the kidneys. For example, the lower extremities are also implicated, which may confer ancillary sequelae on kidneys during reperfusion [59, 60]. The aim of this study was therefore to determine whether systemic TEMPOL administration in rats subjected to moderate renal I/R (30 min ischemia and 90 min reperfusion) induced by aortic clamping instead of suprarenal artery clamping also improves (micro)vascular hemodynamic and oxygenation parameters in the acute reperfusion phase [10]. First, the renal I/R model was validated in terms of oxidative stress, tissue injury, and inflammation parameters, after which a panel of (micro)vascular hemodynamic and oxygenation parameters was assessed in real time following vehicle delivery or TEMPOL treatment. The main findings were that TEMPOL (1) reduced the extent of iNOS activation, tissue injury, and inflammation, (2) down modulated renal vascular resistance to near-baseline levels in the early (15 min) and late (90 min) reperfusion phase, and (3) improved microvascular oxygen tension in the renal cortex and medulla at 90 min reperfusion but not renal oxygen delivery, consumption, and extraction ratio. Overall, the protective effects of TEMPOL were not as profound as was the case in AKI induced by renal artery clamping, which generally was associated with less sizeable alterations in the tested parameters compared to aortic clamping.

**Figure 2.**
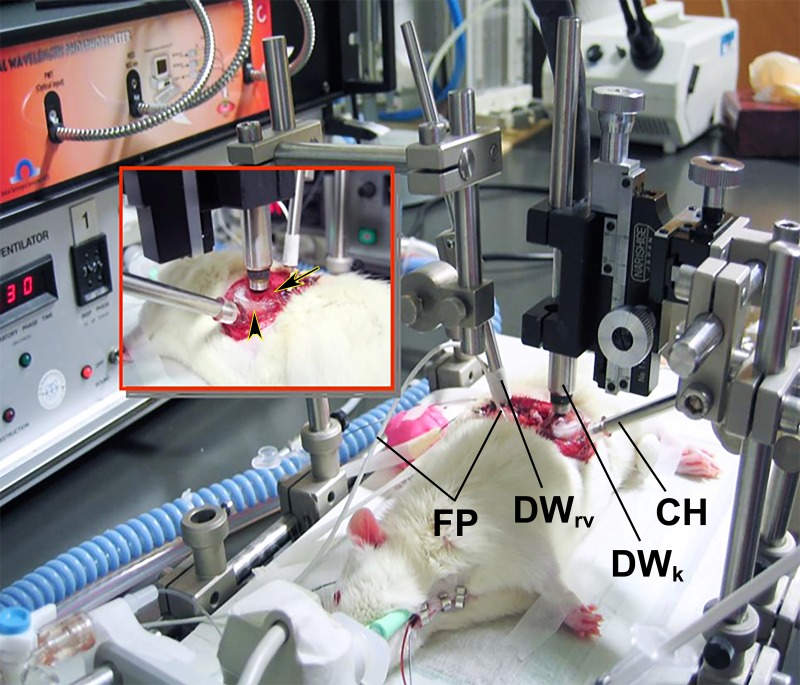
Rat anesthesia, ventilation, sampling, and monitoring setup. The DWrv designates the dual wavelength phosphorimetry probe on the renal vein. The DWk refers to the dual wavelength phosphorimetry probe placed on the kidney. CH signifies the kidney cup holder and FP refers to the ultrasonic flow probe. Insert: rear perspective view of the mobilized left kidney (arrow) positioned in the kidney cup (arrowhead) through the 4-cm dorsal incision.

## 2. Materials and methods

### 2.1. Animals

The animal experiments were approved by the animal ethics committee of the Academic Medical Center of the University of Amsterdam (protocol # DFL83) and animals were treated in accordance with the *Guide for the Care and Use of Laboratory Animals* (NRC 2011). The study was performed with 24 male Wistar rats (Harlan Laboratories, Horst, the Netherlands) with a mean body weight of 348 ± 41 g.

### 2.2. Anesthesia and surgical procedures

Rats were anesthetized by intraperitoneal injection of a mixture of 100 mg/kg ketamine (Nimatek, Eurovet, Bladel, the Netherlands), 0.5 mg/kg dexmedetomidine (Dexdomitor, Pfizer, New York, NY), and 0.05 mg/kg atropine sulfate (Centrafarm, Etten-Leur, the Netherlands). Following a tracheotomy the animals were mechanically ventilated at a FiO_2_ of 0.4. The ventilator settings were adjusted to maintain an end-tidal partial pressure of carbon dioxide (pCO_2_) between 30 and 35 mmHg and an arterial pCO_2_ between 35 and 40 mmHg. Body temperature was maintained at 37 ± 0.5 °C using a heating pad. The surgical field was covered with a humidified gauze compress throughout the entire experiment to prevent desiccation of the exposed tissues.

Blood vessels were cannulated with polyethylene catheters (outer Ø = 0.9 mm, B. Braun Melsungen, Melsungen, Germany). A catheter was inserted into the right carotid artery and connected to a pressure transducer to monitor mean arterial blood pressure (MAP). The right jugular vein was cannulated for continuous infusion of Ringer’s lactate (Baxter Healthcare, Deerfield, IL) at a rate of 15 mL/kg/h and of maintenance anesthesia (50 mg/kg/h ketamine dissolved in Ringer’s lactate, 5 mL/kg/h). The right femoral artery was cannulated for blood sampling and the right femoral vein was cannulated for TEMPOL (400 μmol/kg, dissolved in 0.9 % NaCl) and Oxyphor G2 (6 mg/kg, dissolved in 0.9% NaCl) administration and fluid resuscitation. The left ureter was isolated, ligated, and cannulated with a polyethylene catheter for urine collection and quantification of urine production.

The left kidney was exposed via a ~4-cm incision in the left dorsal flank, decapsulated, and immobilized in a custom-built 3D-printed kidney cup (Lucite International, Hampshire, UK, Figure 2). Renal vessels were carefully separated while preserving the nerves and adrenal gland.

### 2.3. Monitoring of renal blood flow and oxygenation

A perivascular ultrasonic transient time flow probe was placed around the left renal artery (model 0.7 RB, Transonic Systems, Ithaca, NY) and connected to a flow meter (model T206, Transonic Systems) (Figure 2) to continuously measure renal blood flow (RBF).

A detailed description of the phosphorimetry method can be found in [61]. After the surgical procedure, one optical fiber was placed 1 mm above the decapsulated kidney and another optical fiber was placed 1 mm above the renal vein to measure renal microvascular and venous oxygenation, respectively (Figure 2). A small piece of aluminum foil was placed on the dorsal side of the renal vein to prevent phosphorescence signal spillover from underlying tissues during venous partial pressure of oxygen (pO_2_) measurements.

### 2.4. Experimental protocol

The animals were randomly allocated to one of four groups (N = 6 per group). In the first group (‘Ctrl’), animals were sham-operated but not subjected to I/R. In the second group (‘Ctrl + TEMPOL’), rats underwent a sham operation and were administered TEMPOL at 200 μmol/kg/h during 105 min. This dose was validated in our previous study [58]. In the third group (‘I/R’), the animals underwent a 30-min aortic cross-clamping (just below the superior mesenteric artery), rendering both kidneys ischemic, followed by 90 min of reperfusion. In the fourth group (‘I/R + TEMPOL’), rats were given TEMPOL for 15 min prior to ischemia (200 μmol/kg bolus) and during the entire reperfusion phase (200 μmol/kg/h, total infusion time of 90 min). It should be noted that sham-operated animals underwent the same procedures as described in sections 2.2, 2.3, and above except for the cross-clamping of the aorta.

At the end of the experiment the animals were sacrificed by intravenous administration of 1 mL of 3 M potassium chloride. The I/R-subjected kidney was excised for oxidative stress assays (section 2.8) and histological analysis (section 2.9).

### 2.5. Phosphorimetric measurement of renal microvascular and venous PO_2_

Systemically administered Oxyphor G2 (tetra-(4-carboxyphenyl) benzoporphyrin, Oxygen Enterprises, Philadelphia, PA) was used for the measurement of renal oxygenation. Oxyphor G2 binds to albumin and therefore remains confined to the vasculature. The probe has two excitation peaks (λ_ex1_ = 440 nm, λ_ex2_ = 632 nm) and one emission peak (λ_em_ = 800 nm). These optical properties allow near-simultaneous phosphorescence lifetime measurements in the microcirculation of the kidney cortex (CµPO_2_) and the outer medulla (MµPO_2_) due to different optical penetration depths of the excitation light. The local oxygen tension can be extrapolated from the degree of phosphorescence quenching (i.e., shortening of phosphorescence lifetime) by oxygen. The linear relationship between reciprocal phosphorescence lifetimes and oxygen tension, derived from the Stern-Volmer equation, allows quantitative analysis of μPO_2_ [61].

Oxyphor G2 was infused (6 mg/kg during 5 min) 30 min before the start of phosphorimetry (i.e., before ischemia induction). Renal microvascular PO_2_ (µPO_2_) and renal venous PO_2_ (rvPO_2_) were measured at baseline, 15 min of reperfusion (R15), and 90 min of reperfusion (R90). In the non-I/R groups, these parameters were measured at 15 min and 90 min during continuous TEMPOL administration. For the measurement of µPO_2_ and rvPO_2_, dual wavelength phosphorimeters (λ_ex_= 440 and 632 nm) were used (Figure 2).

### 2.6. Calculation of derivative oxygenation parameters and renal vascular resistance

Renal oxygen delivery (DO_2ren_, mL/min) was calculated by RBF × arterial oxygen content (C_a_O_2_ = 1.31 × hemoglobin × S_a_O_2_) + (0.003 × P_a_O_2_), where S_a_O_2_ is arterial oxygen saturation and P_a_O_2_ is arterial partial pressure of oxygen. Renal oxygen consumption (VO_2ren_, mL·min^-1^·g^-1^) was calculated as RBF × (C_a_O_2_ – C_v_O_2_), where renal venous oxygen content (C_v_O_2_) was calculated as (1.31 × hemoglobin × S_rv_O_2_) + (0.003 × rvPO_2_). The S_rv_O_2ren_ was calculated using the Hill equation with P50 = 37 Torr (4.9 kPa) and Hill coefficient = 2.7. The renal oxygen extraction ratio (O_2_ER_ren_, %) was calculated as VO_2ren_ / DO_2ren_ × 100%. An estimation of the renal vascular resistance (RVR, dynes·s^-1^·cm^-2^) was made according to (MAP/RBF) × 100.

### 2.7. Blood gas, acid-base balance, and lactate analysis from blood samples

Arterial blood samples (0.5 mL) were drawn from the femoral artery at baseline, R15, and R90. The same volume of balanced colloid solution (VOLUVEN, Fresenius Kabi, Bad Homburg, Germany), was infused to correct for the very mild transient hypovolemia. Blood gas values, hemoglobin concentration, hemoglobin oxygen saturation, and acid-base balance were determined directly after sampling (ABL 505 blood gas analyzer, Radiometer, Copenhagen, Denmark).

Plasma lactate levels were assayed in samples acquired at baseline and at R90 by an enzymatic colorimetric method (modular P800 automatic analyzer, Roche Diagnostics, Basel, Switzerland).

It should be noted that urine-based kidney function parameters (urine production, creatinine clearance rate / glomerular filtration rate, and renal sodium reabsorption [58]) could not be determined because of I/R-induced anuria in all groups.

### 2.8. Determination of renal oxidative stress

Tissue malondialdehyde (MDA) levels were used as a measure of lipid peroxidation to determine the extent of oxidative stress. Kidneys were homogenized in ice-cold 5 mM sodium phosphate buffer. The homogenates were centrifuged at 12,000 ×g for 15 min at 4 °C and the supernatant was used for MDA determination by tandem mass spectrometry [62-64] in accordance with [65]. The level of lipid peroxides was expressed as micromoles of MDA per milligram of protein (Bradford assay).

MDA was quantified using a Quattro Premier XE tandem mass spectrometer (Waters, Milford, MA) with an Acquity sample manager and an Acquity binary solvent manager. MDA and MDA-d2 were separated on a Supelco LC-18DB column (250-mm length, 4.6-mm diameter, 5-µm particles) using an isocratic run from 50% acetonitrile, 50% water, and 0.2% acetic acid. The flow rate was set to 1 mL/min with a total run time of 10 min. Both compounds were detected and quantified by MRM acquisition in positive electrospray ionization mode, using the transitions m/z 235 > 159 for MDA and 237 > 161 for MDA-d2.

### 2.9. Immunohistochemistry

Kidney tissue was fixed in 10% formalin and embedded in paraffin. Kidney sections (5 μm) were deparaffinized with xylene and rehydrated with decreasing grades of ethanol and finally with water. Antigen retrieval was accomplished by microwaving the sections in citrate buffer (pH = 6.0) for 10 min at 800 W. Sections were cooled for 20 min at room temperature (RT) and rinsed with MilliQ. The section margins were marked with a PAP pen. Endogenous peroxidase activity was blocked with 3% H_2_O_2_ for 10 min at room temperature (RT), after which the sections were rinsed with MilliQ and PBS. Blocking reagent (TA-125-UB, Lab Vision, Fremont, CA) was applied to each slide for 5 min at RT in a humidified chamber. The sections were incubated overnight at 4 °C with rabbit anti-mouse inducible nitric oxide synthase (iNOS) (iNOS rabbit PabNeomarker, RB-1605-P, Lab Vision), interleukin (IL)-6 (product # 6672, Abcam, Cambridge, UK), myeloperoxidase (MPO) (MPO rabbit RB-373-A, Thermo Fisher Scientific, Waltham, MA), neutrophil gelatinase-associated lipocalin (NGAL) (product # 41105, Abcam), and liver-type fatty acid binding protein (L-FABP) (product # HP8010, Hycult Biotech, Uden, the Netherlands). Antibodies were diluted in a large volume of UltrAb Diluent (product # TA-125-UD, Lab Vision) at a 1:100 dilution. The sections were washed thrice in PBS (5 min per washing step) and incubated for 30 min at RT with biotinylated goat anti-rabbit secondary antibodies (product # TR-125-BN, Lab Vision) [66]. Next, the streptavidin peroxidase label reagent (product # TS-125-HR, Lab Vision) was applied for 30 min at RT in a humidified chamber. The colored product was developed by incubation with AEC (product # TA-007-HAC, Lab Vision). The slides were counterstained with hematoxylin and mounted in glycerol gelatin after being washed in distilled water. Both the intensity and the distribution of antigens were scored.

For each sample, a histological score (HSCORE) value was derived by summing the percentages of cells that stained at each intensity multiplied by the weighted intensity of the staining (HSCORE = S P_i_ (*i*+1), where *i* is the intensity score and P_i_ is the corresponding percentage of the cells [67]). MPO was scored in 30 selected glomeruli and peritubular areas (binary scoring system; 1 if leukocytes were observed in the glomerulus and 0 if not).

### 2.10. Statistical analysis

The decay curves of phosphorescence intensity were analyzed in LabVIEW (National Instruments, Austin, TX) as described in [61]. Statistical analysis was performed using GraphPad Prism (GraphPad Software, San Diego, CA). Intragroup differences between ordinal variables were analyzed using a two-way ANOVA with a Bonferroni post-hoc test (different time points in the same group). Intergroup differences were analyzed with a repeated measures ANOVA using a Tukey post-hoc test (same time points in different groups). A *p*-value of ≤ 0.05 was considered statistically significant. Data are reported as mean ± standard deviation (SD).

## 3. Results and Discussion

### 3.1. Characterization and validation of the ischemia/reperfusion acute kidney injury model

To validate our I/R AKI model and the pharmacodynamic effects of TEMPOL in juxtaposition to the results obtained in literature and our parallel study with renal artery occlusion [58], the degree of iNOS expression, oxidative stress, kidney damage, and inflammation were analyzed first in rats subjected to 30 min of renal ischemia followed by 90 min of reperfusion. This I/R regimen has been standardly employed as a model for AKI [68, 69]. The renal hemodynamics and oxygenation parameters, which constituted the main focus of the study, are presented and discussed following the model validation.

#### 3.1.1. TEMPOL reduces I/R-induced renal iNOS and IL-6 expression

Inducible nitric oxide synthase (iNOS), which produces NO from L-arginine upon activation, is replete in macrophages that have infiltrated the injured kidney as well as tubular epithelium [70] but not parenchymal cells [71]. iNOS is activated under oxidative stress conditions via redox-sensitive nuclear factor kappa-light-chain-enhancer of activated B cells (NF-κB) [72] and by pro-inflammatory cytokines such as tumor necrosis factor alpha (TNF-α), IL-1, -6, -12, and -18, as well as interferon-γ [73-75], all of which are released during renal I/R [73, 76].

**Figure 3.**
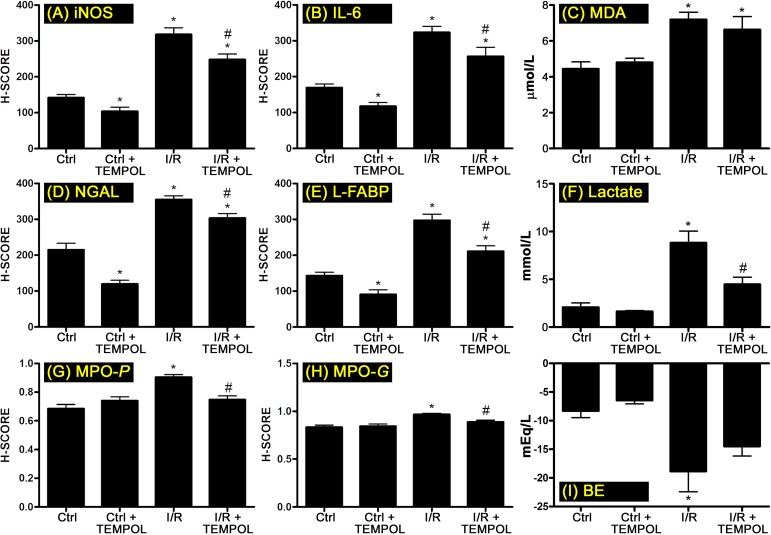
Summary of iNOS activation (A), oxidative stress (C), kidney damage (D-F), inflammation (B, G, H),and base excess (I) following sham operation or 30 min ischemia and 90 min reperfusion in rat kidneys. In the TEMPOL groups, rats received TEMPOL at 200 μmol/kg/h during 105 min (in the I/R + TEMPOL group, 15 min before ischemia and during 90 min of reperfusion). Abbreviations: iNOS, inducible nitric oxide synthase; IL-6, interleukin-6; MDA, malondialdehyde; NGAL, neutrophil gelatinase-associated lipocalin; L-FABP, renal liver-type fatty acid binding protein; MPO-*P*, peritubular myeloperoxidase; MPO-*G*, glomerular myeloperoxidase; BE, base excess. Values are represented as mean ± SD, *p < 0.05 vs. Ctrl; ^#^*p* < 0.05 vs. I/R. Abbreviations: I/R, ischemia/reperfusion.

In the renal cortex, iNOS expression was increased in the I/R group compared to control (Figure 3A), which is consistent with earlier reports [77]. This trend was mirrored by increased IL-6 immunostaining (Figure 3B), also occurring in accordance with previous findings [78] and thereby validating our I/R AKI model. The administration of TEMPOL before ischemia and during reperfusion ameliorated the extent of iNOS expression (Figure 3A) and decreased IL-6 levels at a similar rate relative to iNOS (Figure 3B). The same trend was observed in TEMPOL-treated rats that did not undergo I/R compared to baseline levels (Ctrl group, Figure 3A and B), most likely because the surgical procedures alone led to a mild sterile immune response.

An iNOS-reductive effect has not been described before for TEMPOL. Although not confirmed experimentally, this effect is likely related to the superoxide scavenging properties of TEMPOL, as a result of which hydrogen peroxide production and corollary NF-κB activation [79, 80] and thus iNOS hyperactivation and NO overproduction are suppressed. Our parallel study with suprarenal artery clamping as a model for I/R AKI [58] demonstrated a considerable drop in renal NO levels at 90 min reperfusion (reflecting an increase in peroxynitrite formation). The intrarenal NO concentration was restored to pre-I/R levels by TEMPOL pretreatment in that model, implying that peroxynitrite formation was blocked due to reduced superoxide production. Although NO levels were not directly measured in this study, the post-ischemic increase in tissular iNOS (I/R group, Figure 3A) strongly suggests a burst in NO production that, in the absence of excessive superoxide and hence peroxynitrite generation (I/R + TEMPOL group) [29], conferred its well-established protective effects on kidney function and viability [81-86]. Thus, inhibition of the pro-inflammatory response and the cell-protective effects by TEMPOL may translate to reduced NOS uncoupling and increased NO bioavailability.

#### 3.1.2. TEMPOL does not alleviate lipid peroxidation in renal tissue in the acute reperfusion phase following aortic clamping-induced renal I/R

At low concentrations, NO causes dilation of the renal (micro)circulation [87] that is associated with improved renal function after I/R [88]. During all phases of I/R, however, superoxide is excessively produced intracellularly and extracellularly by different molecular and cellular sources [10] and reacts with NO to form peroxynitrite [34, 35] and tertiary radical derivatives that induce oxidative damage in the cellular and vascular compartment [9, 29]. As shown in Figure 3C, lipid peroxidation (MDA) in renal tissue was exacerbated by I/R but not reduced by TEMPOL. A TEMPOL-mediated reduction in lipid peroxidation has been reported before in a rat renal I/R model employing 45 min ischemia and 6 h reperfusion [54], which is a more severe I/R model where sufficient time was provided for the manifestation of oxidative/nitrosative stress in the chronic reperfusion phase [10].

As recently advocated [89], pharmacological interventions should be tested in both mild and severe I/R injury models. When the aortic occlusion was performed more proximally to the kidney (i.e., suprarenal artery instead of the abdominal aorta just below the superior mesenteric artery), MDA levels were significantly reduced by prior TEMPOL administration (200 µmol/kg/k for 15 min prior to ischemia but *not* during the reperfusion phase) [58]. This difference in outcome implies that the concomitant manifestation of lower limb ischemia contributed to post-ischemic oxidative stress in the kidneys and abrogated the antioxidant efficacy of TEMPOL, at least at this administered dose.

It should further be noted that the chain propagating redox reaction that is characteristic of lipid peroxidation is mainly deterred by lipophilic antioxidants [29], whereas TEMPOL, with a computed logP of 0.9 (PubChem), is hydrophilic and may therefore not intercalate in cell- and subcellular membranes to prevent lipid peroxidation. Instead, TEMPOL may confer anti-oxidant protection in plasma and the cytosol and thereby prevent superoxide-derived secondary and tertiary ROS/RNS from attacking membrane constituents and reduce renal dysfunction and I/R injury through its superoxide scavenging activity [48, 90].

#### 3.1.3. TEMPOL reduces ischemia/reperfusion-mediated acute kidney injury

NGAL and L-FABP are biomarkers of acute kidney injury [91, 92]. After 90 min of reperfusion, both NGAL and L-FABP were increased in kidney tissue (Figure 3D and E). An increase in NGAL following I/R in rats is in agreement with literature [93, 94]. No studies could be retrieved for L-FABP in terms of renal I/R injury in rats, although urinary L-FABP levels were found to be predictive of the severity of AKI in mice [95]. TEMPOL treatment resulted in decreased levels of these biomarkers in both control and I/R-subjected rats (Figure 3D and E).

Similar results were obtained in terms of plasma lactate (Figure 3F), although this biomarker may have confounding causes such as defects in blood supply (type A lactic acidosis) and perturbed aerobic respiration/electron transport chain functionality in mitochondria (type B lactic acidosis) [96]. Both are pertinent to renal I/R AKI but do not reflect direct (histological) tissue injury, in which case lactate is liberated from dying and dead cells. Moreover, lactate is removed from the circulation via renal clearance (mainly under conditions of hyperlactatemia, Figure 3F) and metabolism [97]. Renal contribution to lactate metabolism is influenced by renal mitochondrial metabolism (which seemed to be unperturbed based on the O_2_ER_ren_ data) and glomerular filtration (which was absent). The lactate data may therefore also have been skewed by mainly the fact that the I/R-subjected kidneys were hypoperfused (section 3.2.1) and anuric in all groups (data not shown). This would shift the lactate clearance to the liver [97], despite the fact that the prevailing acidosis during reperfusion (Supplemental Figure 1) generally improves renal removal of lactate and inhibits its hepatic clearance [97]. Finally, the extent of contribution of the hind limb ischemia to lactate kinetics and disposition is elusive [98]. TEMPOL was not able to restore urine production (i.e., primary kidney function), which was not the case in our parallel study employing a less severe I/R model with suprarenal artery clamping [58]. The anuria was likely caused by the significantly reduced MAP (Table 1). TEMPOL did normalize the acidosis (Supplemental Figure 1), most likely due to its beneficial effects on microcirculatory oxygenation in the hind limb and on kidney function, which is related to the production of bicarbonate.

As alluded to previously, the renal damage- and functionameliorating effects of TEMPOL are well-established. This is partly due to its inhibition of iNOS activation (Figure 3A) inasmuch as the extent of iNOS activation and NO production are proportional to the degree of apoptotic tubular and glomerular cell death [99] and AKI [84, 85]. Taken together, these data validate the AKI component of our I/R model and connect iNOS/NO to oxidative stress and AKI.

#### 3.1.4. TEMPOL reduces peritubular and glomerular neutrophil influx and inflammation

TEMPOL has been shown to reduce post-ischemic inflammatory signaling in a rat [54] and mouse[57] model of renal I/R and a Guinea pig model of gallbladder I/R [56]. Oxidative stress and cell death during I/R lead to highly pleiotropic immune signaling and sterile inflammation [9], which was confirmed in our model on the basis of the IL-6 (Figure 3B) and MPO data (Figure 3G, H). In addition to reducing tissular IL-6 levels following I/R (Figure 3B), TEMPOL treatment also reduced the degree of neutrophil influx into peritubular regions (Figure 3G) as well as glomeruli (Figure 3H), an effect that has been reported previously in the I/R AKI setting [54], albeit in a more rigorous damage model. As depicted in Figure 1 and described in section 1, a reduction in sterile inflammation has beneficial implications on the pathogenesis of AKI. Accordingly, amelioration of pro-inflammatory signaling by TEMPOL (Figure 3B, G, H) was associated with reduced AKI (Figure 3D-F).

### 3.2. Renal hemodynamic and oxygenation alterations in response to moderate ischemic injury

In terms of the parameters assessed in section 3.1, the increase in iNOS activation, oxidative stress, kidney damage, and inflammation during the acute reperfusion phase was generally moderate in our AKI model of 30 min ischemia and 90 min reperfusion compared to studies employing longer I/R durations. The present model was deliberately employed because (a) it is more clinically representative than our previous work using renal artery clamping [58], (b) it has been standardized for I/R AKI in numerous other studies (e.g., [68, 69]), and (c) we did not want to exceed the maximum damage threshold beyond which intervening with TEMPOL would no longer be useful [89]. Moreover, as has been explained in detail in [8], iNOS hyper activation, inflammation, and deleterious changes in mitochondrial metabolism and redox balance have a disproportionally detrimental effect on renal hemodynamics and oxygenation and consequently on primary kidney function (i.e., electrolyte homeostasis mediated by the electrolyte transporter Na^+^/K^+^-ATPase, which is a heavy oxygen-consuming process in the kidney[8]) and viability, particularly in the outer medulla and corticomedullary junction [100]. Kidneys are particularly sensitive to hypo-oxygenation (I/R) and oxidative/nitrosative stress for several reasons. First, the renal vascular anatomy and physiology (inherently low medullary blood flow and oxygen supply) precludes adequate management of hypoxic/anoxic conditions. Second, a large fraction of the available oxygen is used to produce ATP for the electrolyte transporter Na^+^/K^+^-ATPase, as a result of which a relatively minor fraction of ATP is available to sustain cell viability. Third, excessive NO and ROS/RNS production during I/R causes mitochondrial dysfunction and an immediate reduction in renal oxygenation [8, 36, 58], further debilitating medullary and cortical oxygen availability [58] at the cellular level and therefore primary kidney function and viability. Accordingly, by implementing a moderately injurious ischemia time we could control the parameters addressed in section 3.1 so as to prevent disproportionally severe hypoxia and irreversible kidney damage, to which the hind limb I/R was also expected to contribute [59, 60].

#### 3.2.1. TEMPOL reduces increased renal vascular resistance following ischemia/reperfusion but has no effect on other systemic and local hemodynamic parameters

The hemodynamic parameters at baseline, R15, and R90 are listed in Table 1 and show that I/R was associated with a drop in MAP and RBF and an increased RVR. The values in the I/R group clearly demonstrate the more profound impact of aortic clamping on systemic and renal hemodynamic parameters com pared to clamping of only the renal artery [58]. Illustratively, the MAP in this study decreased by 38% and 47% at R15 and R90, respectively, versus a decrease of merely 7% and −2%, respectively, in case of renal artery clamping [58]. These data might be explained by the suprarenal aortic occlusion, which also blocks the inferior mesenteric artery blood supply and may lead to sepsis-induced hypotension due to increased bacterial translocation into blood stream. The RBF declined by 76% (R15) and 78% (R90) after aortic occlusion, in line with previous reports [16], which occurred at approximately half these rates (39% and 37%, respectively) following renal artery occlusion [58]. The RVR increased by 313% (R15) and 303% (R90) compared to 154% and 162%, respectively, following renal artery occlusion [58].

**Table 1.** Hemodynamic parameters (mean ± SD) at baseline and at 15 min (R15) and 90 min (R90) reperfusion. A superscript ‘C’ indicates that *p* ≤ 0.05 vs. the control group (Ctrl) and a superscript ‘I’ signifies that p ≤ 0.05 vs. The ischemia/reperfusion group (I/R).

	Baseline	R15	R90
MAP [mmHg]			
Ctrl	93.1 ± 10.7	86.1 ± 5.1	69.0 ± 6.6
Ctrl + TEMPOL	92.1 ± 8.1	87.6 ± 8.5	80.8 ± 3.6
I/R	99.0 ± 12.6	53.0 ± 18.7^C^	36.5 ± 10.5^C^
I/R + TEMPOL	86.3 ± 10.5	60.6 ± 16.9^C^	44.3 ± 5.9^C^
RBF [mL·min^-1^]			
Ctrl	4.3 ± 0.7	3.8 ± 1.2	3.2 ± 0.4
Ctrl + TEMPOL	4.2 ± 0.7	3.6 ± 1.0	3.6 ± 1.3
I/R	4.7 ± 1.5	0.9 ± 0.7^C^	0.7 ± 0.3^C^
I/R + TEMPOL	3.9 ± 0.3	1.9 ± 0.7^C^	1.4 ± 0.3^C^
RVR [dyn·s·cm^2^]			
Ctrl	2217 ± 418	2390 ± 671	2169 ± 278
Ctrl + TEMPOL	2208 ± 447	2598 ± 835	2438 ± 800
I/R	2276 ± 801	7485 ± 4316^C^	6581 ± 3404^C^
I/R + TEMPOL	2238 ± 391	3399 ± 1540^I^	3378 ± 928^I^

Abbreviations: R15, reperfusion at 15 min; R90, reperfusion at 90 min; MAP, mean arterial pressure, RBF, renal blood flow; RVR, renal vascular resistance.

The increased RVR is attributable to constriction of renal microvasculature, which can have multiple causes such as an I/R-induced imbalance between vasodilatory and vasoconstrictive mediators and compression of peritubular capillaries due to interstitial edema [8]. iNOS, which was considerably elevated during I/R (Figure 3A), plays an instrumental role in post-ischemic modulation of vascular tonicity [101, 102]. These data together attest to the fact that the clinically representative I/R model, which encompasses supraregional effectors (e.g., hind limb I/R), has a more deleterious impact on AKI than when only renal artery occlusion is applied.

The more prolific renal damage profile seemed to be inversely proportional to TEMPOL’s pharmacodynamic efficacy, even when the cumulative administered dose was considerably higher compared to that used in the renal artery occlusion study [58] due to the continuous administration throughout the 90-min reperfusion phase. The MAP and RBF were not alleviated by TEMPOL (Table 1), at least not to a statistically significant degree. In the renal artery clamping study [58], TEMPOL evidently exerted no effect on the MAP (which was unimpaired by I/R) but normalized the RBF to Ctrl levels at both R15 and R90 [58]. Inasmuch as glomerular filtration rate declines in concordance with declining RBF and the MAP (Table 1) [103-105], it can be inferred that I/R in the present model was associated with strongly deteriorated glomerular filtration (i.e., renal function), supported by the absence of urine production (section 3.1.3), most likely as a result of systemic hemodynamics (MAP). However, we demonstrated that administration of TEMPOL resulted in a significant improvement in renal vascular resistance as a result of vasodilatation in the renal vascular bed and increased NO bioavailability. Following this line of reasoning, it can be concluded that TEMPOL was partially effective in safeguarding primary renal function after aortic occlusion-induced I/R.

#### 3.2.2. TEMPOL improves cortical and medullary microvascular oxygenation but not renal oxygen delivery, renal oxygen consumption, and renal oxygen extraction ratio

The renal oxygenation parameters are summarized in Table 2. As with the systemic and hemodynamic variables (Table 1), a similar trend was observed in terms of the change amplitude of parameters in the aortic clamping group versus the renal artery clamping group [58]. Whereas the DO_2ren_ was decreased by 36% (R15) and 41% (R90) after I/R [58] in the latter group, the decreases following aortic clamping were 76% (R15) and 83% (R90). The rate at which the DO_2ren_ decreased was very comparable to the rate at which the RBF decreased (Table 1 and [58]). Furthermore, the post-ischemic VO_2ren_ was unaffected by renal artery clamping [58] yet considerably compromised by aortic clamping (−79% at R15 and −82% at R90 compared to Ctrl). These events were accompanied by reduced CμPO_2_ (26% at R15 and 52% at R90) and MμPO_2_ (32% at R15 and 61% at R90), that, again, encompassed more profound changes in microvascular oxygenation than observed during renal artery occlusion-induced I/R (CμPO_2_, 16% at R15 and 29% at R90; MμPO_2_, 7% at R15 and 20% at R90) [58]. TEMPOL treatment only partially restored the drop in CμPO _2_ (↑ by 42%) and MμPO_2_ (↑ by 68%) at R90, but had otherwise no significant effect on these parameters at R15 or on the DO_2ren_ and VO_2ren_ altogether, despite the fact that these values more than doubled at both reperfusion time points in the I/R + TEMPOL group compared to the I/R group. Provided that a large fraction of oxygen is used to support the functionality of the Na^+^/K^+^-ATPase [8], the observation that O_2_ER_ren_ (VO_2ren_ / DO_2ren_) remained unaltered following aortic clamping-induced I/R (Table 2) can be biochemically and physiologically accounted for, at least partially. The rise in intrarenal iNOS (Figure 3A) coincides with a burst in local NO production [33] that, in a milieu of concomitant superoxide hyperproduction [23-25,58], results in peroxynitrite formation [34, 35] and consequent decrease in Na^+^/K^+^-ATPase activity [36]. The perturbed Na^+^/K^+^-ATPase activity translates to lower oxygen demand in the kidneys (i.e., lowered demand for ATP) and hence a decrease in VO_2ren_ that, under conditions of reduced RBF (Table 1), CμPO_2_, MμPO_2_, and DO_2ren_ (Table 2), ultimately has no net effect on O_2_ER_ren_.

**Table 2.** Renal oxygenation parameters at baseline and at 15 min (R15) and 90 min (R90) reperfusion. All data are presented as mean ± SD. ^C^*p* < 0.05 vs. Ctrl; ^I^*p* < 0.05 vs. I/R.

	Baseline	R15	R90
DO_2ren_ [mL O_2_/min]			
Ctrl	3.55 ± 0.63	3.01 ± 1.12	2.46 ± 0.42
Ctrl + TEMPOL	3.68 ± 0.61	3.00 ± 0.85	2.98 ± 0.97
I/R	3.81 ± 1.23	0.72 ± 0.57^C^	0.43 ± 0.32^C^
I/R + TEMPOL	3.17 ± 0.40	1.56 ± 0.69^C^	0.99 ± 0.28^C^
VO_2ren_ [mL O_2_/min/g]			
Ctrl	1.58 ± 0.28	1.47 ± 0.54	1.35 ± 0.26
Ctrl + TEMPOL	1.72 ± 0.28	1.52 ± 0.50	1.60 ± 0.56
I/R	1.36 ± 0.47	0.31 ± 0.23^C^	0.24 ± 0.23^C^
I/R + TEMPOL	1.31 ± 0.24	0.75 ± 0.40^C^	0.58 ± 0.17^C^
CμPO_2_ [mmHg]			
Ctrl	81.6 ± 7.6	68.9 ± 14.4	70.3 ± 11.3
Ctrl + TEMPOL	89.5 ± 2.7	84.4 ± 6.1^C^	69.1 ± 6.3
I/R	84.6 ± 3.2	50.9 ± 14.3^C^	33.6 ± 7.6^C^
I/R + TEMPOL	89.5 ± 9.4	55.3 ± 8.4^C^	47.7 ± 5.4^C,I^
MμPO_2_ [mmHg]			
Ctrl	64.3 ± 8.0	59.8 ± 9.9	56.8 ± 8.2
Ctrl + TEMPOL	68.3 ± 9.0	62.3 ± 7.1	57.0 ± 6.8
I/R	65.8 ± 4.1	40.7 ± 10.8^C^	21.9 ± 7.4^C^
I/R + TEMPOL	59.7 ± 7.9	43.6 ± 5.4^C^	36.7 ± 4.8^C,I^
O_2_ER_ren_ [%]			
Ctrl	44.6 ± 3.6	49.3 ± 4.1	55.1 ± 6.7
Ctrl + TEMPOL	46.8 ± 3.2	50.1 ± 5.0	53.6 ± 3.5
I/R	36.2 ± 8.0	44.7 ± 7.9	50.0 ± 21.4
I/R + TEMPOL	40.9 ± 2.6	46.2 ± 7.3	58.5 ± 8.0

Abbreviations: R15, reperfusion at 15 min; R90, reperfusion at 90 min; DO_2ren_, renal oxygen delivery (the total amount of oxygen delivered to the tissues per minute, irrespective of the distribution of blood flow); VO_2ren_, renal oxygen consumption (total amount of oxygen removed from the blood due to tissue oxidative metabolism per minute); CμPO_2_, microvascular oxygen tension in the renal cortex; MμPO_2_, microvascular oxygen tension in the renal medulla; O_2_ER_ren_, renal oxygen extraction ratio (VO_2ren_ / DO_2ren_).

The O_2_ER_ren_ is essentially a measure of how well metabolic demand (ATP) is aligned with metabolic supply (oxygen). Inasmuch as the DO_2ren_ reflects oxygen delivery to tissue irrespective of the distribution of blood flow, the O_2_ER_ren_ signifies the degree to which oxygen is uncoupled from hemoglobin and used in aerobic respiration in cells that comprise the perfused tissue. Somewhat surprisingly, post-ischemic renal cells did not exhibit an increased predilection for oxygen (Table 2, O_2_ER_ren_) under conditions of hypoperfusion (Table 1, RBF) and reduced μPO_2_ (Table 2). It is not illogical to expect that the oxygen-driven metabolic rate in viable mitochondria [23] of hypoxia-subjected cells would be increased to compensate for the perturbed aerobic metabolism in oxidatively compromised mitochondria [46, 106]. Analogous compensatory effects have been described in other I/R settings, such as reactive hyperemia in upper extremities of human subjects [107] and post-ischemic NADH hyperoxidation in rat livers [21]. However, in this case it seems that the metabolic demands of renal cells were met, even at the clearly perturbed oxygenation parameters (Table 2). In that respect, it has been reported that kidneys are quite able to maintain a stable O_2_ER over a wide range of conditions [108].

Two possible explanations are that, one the one hand, the damage to renal cells was not as profound as the degree of Na^+^/K^+^-ATPase shutdown, which would account for the considerable reduction in VO_2ren_ without notable changes in O_2_ER_ren_, especially since cessation of Na^+^/K^+^-ATPase activity would reallocate the ATP to other vital cell metabolic processes (e.g., survival and damage repair). Such a scenario implies that the impact of the hind limb I/R was most profound on the manifestation of renal pathophysiology. On the other hand, the renal cells were damaged to such an extent [8] that aerobic respiration had mainly ceased in the kidney as a result of mitochondrial dys-/non-function [8, 36, 58]. This is supported by the 79-82% drop in VO_2ren_ during the 90-min reperfusion phase (Table 2), and the prevailing metabolic acidosis, as evidenced by the combination of elevated plasma lactate levels (Figure 3F), reduced pH (Supplementary Figure 1), relatively stable pCO_2_ values (data not shown), and considerably lowered negative base excess ratio (Figure 3I).

Juxtaposition of our result to literature plead for the first scenario, namely that 30/90 min I/R was in itself not very injurious to the kidney but ultimately led to AKI due to the contribution of hind limb I/R. In a study by de Carvalho et al. [93], rats were subjected to 30-min renal artery clamping, after which NGAL and histological damage were determined in blood samples and by evaluation by a pathologist, respectively, at different reperfusion times. A moderate-to-severe damage profile, characterized by 25-50% tubular necrosis, was noted at 12-h reperfusion, which corresponded to a 3,636% increase in plasma NGAL compared to baseline after only renal artery occlusion [93]. By deduction, the 167% increase in tissue NGAL (Figure 3D) reflects minimal histological damage. Moreover, a reduction in MAP was not observed after renal clamping [58], altogether pleading in favor of the hypothesis that the hind limb I/R and bacterial translocation conferred a more debilitating effect on kidney viability and function than I/R affecting the kidneys only. In that respect, the metabolic acidosis, as alluded to in the second abovementioned hypothesis, may very well have stemmed from chiefly the hind limb I/R and masked the relatively mild direct effects of this I/R regimen on the kidneys. However, additional studies are warranted to examine the efficacy of higher TEMPOL doses and the pathological contribution of separate anatomical compartments to post-ischemic kidney injury.

## 4. Conclusions

In the final analysis, TEMPOL administration at a cumulative dose of 400 μmol/kg before aortic occlusion-induced ischemia and during the early reperfusion phase conferred a protective effect on AKI in terms of renal damage, inflammation, and iNOS production. The beneficial effects of TEMPOL on renal hemodynamics and oxygenation were limited, however, only manifesting themselves at the level of RVR and μPO_2_ but not MAP, RBF, DO_2ren_, VO_2ren_, and O_2_ER_ren_. The main overshadowing elements in the renal pathophysiology were the drop in MAP in consequence to the conjoint hind limb and gut I/R, which led to hypotension and abrogated glomerular filtration (i.e., anuria), and perhaps metabolic acidosis. Although TEMPOL alleviated the metabolic acidosis, the superoxide scavenger exerted no evident beneficial effects on the MAP, which should be accounted for by alternative interventions to ensure sustenance of renal function during clinical procedures that involve aortic (cross-)clamping. Our findings are particularly important for the renal transplantation setting, as these procedures entail aortic clamping as well as stimulation of innate and adaptive immunity following transplantation. A pharmacological role of TEMPOL may therefore be even more limited in this context, and other therapeutics in addition to immunosuppressive drugs should be studied to protect both donor (in case of living donor kidney transplantation) and recipient.
